# Persistence of physical activity in middle age: a nonlinear dynamic panel approach

**DOI:** 10.1007/s10198-013-0518-8

**Published:** 2013-07-17

**Authors:** Narimasa Kumagai, Seiritsu Ogura

**Affiliations:** 1 Faculty of Economics, Kinki University, 3-4-1 Kowakae Higashiosaka, Osaka, 577-8502 Japan; 2 Department of Economics, Hosei University, 4342 Aihara Machida, Tokyo, 194-0298 Japan

**Keywords:** Dynamic panel probit model, Health production, Physical activity, Latent health stock, State dependence, C23, C51, I12

## Abstract

No prior investigation has considered the effects of state dependence and unobserved heterogeneity on the relationship between regular physical activity (RPA) and latent health stock (LHS). Accounting for state dependence corrects the possible overestimation of the impact of socioeconomic factors. We estimated the degree of the state dependence of RPA and LHS among middle-aged Japanese workers. The 5 years’ longitudinal data used in this study were taken from the Longitudinal Survey of Middle and Elderly Persons. Individual heterogeneity was found for both RPA and LHS, and the dynamic random-effects probit model provided the best specification. A smoking habit, low educational attainment, longer work hours, and longer commuting time had negative effects on RPA participation. RPA had positive effects on LHS, taking into consideration the possibility of confounding with other lifestyle variables. The degree of state dependence of LHS was positive and significant. Increasing the intensity of RPA had positive effects on LHS and caused individuals with RPA to exhibit greater persistence of LHS compared to individuals without RPA. This result implies that policy interventions that promote RPA, such as smoking cessation, have lasting consequences. We concluded that smoking cessation is an important health policy to increase both the participation in RPA and LHS.

## Introduction

The negative effects of physical inactivity on health are well known. Unhealthy diets and physical inactivity are the main contributors to overweight and obesity, which are among the leading risk factors for the major non-communicable diseases [1]. Heart disease is a costly outcome of physical inactivity [2].

However, half of Japanese workers are physically inactive because they do not engage in enough physical activity during their leisure time and because jobs are increasingly sedentary in nature. The 2007 National Health and Nutrition Survey in Japan indicated that one out of two males 40–74 years old was likely to develop metabolic syndrome.^1^ In addition, the prevalence of overweight or obesity in Japan (a body mass index of 25 or higher) showed a tendency to increase in males regardless of age group compared with 1997 statistics. The change in the prevalence of overweight or obesity in males aged 50–59 in a decade was 10.2 % points (from 24.1 to 34.3 %), the largest value among the working generations. In contrast, the proportion of regular exercisers among males aged 50–59 was 21.0 %, which was smaller than the value of 24.7 % for females.^2^

The effect in which a past value by itself influences future values of the same process is known as genuine state dependence. To the best of our knowledge, only a few studies have taken into account the importance of both state dependence and unobserved heterogeneity in explaining health outcomes [3–5]. The studies by Contoyannis et al. [3, 4] supported the existence of a certain degree of state dependence in self-assessed health in the UK. Contoyannis et al. [3] presented evidence of persistence in self-assessed health, attributed in part to state dependence, and found that such persistence was stronger among men than among women. They showed that the impact of individual heterogeneity was reduced when state dependence was controlled for and that unobserved heterogeneity accounted for 30 % of the unexplained variation in health. Hernández-Quevedo et al. [5] reported that health limitations had a high state dependence even after controlling for measures of socioeconomic status. Their model conditioned on previous health status and parameterized the unobserved individual effect as a function of the initial period observations on time-varying regressors and health.^3^

Taking into account the unobserved heterogeneity of employed persons, Brown and Roberts [6] examined frequency of participation in physical activity using a generalized random-effects-ordered probit model and revealed that there was a time trade-off among non-market work, market work, and the frequency of participation. They used the method of Mundlak [7], which takes the group means of the time explanatory variables into account in order to remove the time invariant individual effects from the model, thereby allowing for unbiased estimation. However, they did not take into account the contribution of the state dependence to participation in physical activity.

Health depreciation may not be solely a consequence of aging but may also be related to adverse health behavior. In line with Grossman [8], health behavior can be treated as an investment in health. The starting point for an economic analysis of health dynamics is Grossman’s household production model. Grossman’s investment model determines the optimal stock of health in any period.^4^ Under a partial adjustment mechanism, because of adjustment costs to the desired health stock, current health will depend on previous health, and this model can be estimated using longitudinal data [4]. We include the lagged dependent variables in our dynamic empirical models and suggest that these may be viewed as approximating partial adjustment mechanisms. Our models include regular physical activity (RPA) as a representative lifestyle choice. We consider that there may be a direct causal link between lifestyle choices such as RPA and health status.

In this article, we examine the association between participation in RPA and latent health stock (LHS) in a middle-aged population, drawing on the health investment framework of Grossman’s model. We focus on measuring the degree of genuine state dependence in both RPA and LHS. Accounting for state dependence will correct the possible overestimation of the impacts of socioeconomic factors. Estimation results showing that the degree of state dependence of LHS is positive and significant would imply that policy interventions that improve LHS will have lasting consequences over time.

The remainder of this article is structured as follows. In section “Participation in physical activity and latent health stock”, we summarize the studies on participation in physical activity. Following a review of the literature, we describe the characteristics of the longitudinal data used in this study. Data from a large nationwide survey by the Japanese Ministry of Health, Labor and Welfare were used. In longitudinal data (panel data) analysis, it is possible to focus on changes in health behavior occurring in subjects and to make population inferences that are not as sensitive to variations between subjects. In section “Empirical strategy and results”, we present the estimation results of three probit models of middle-aged populations, taking physical health variables into account. Comparing the estimation results of the pooled probit model, random-effects probit model, and dynamic random-effects probit model, we show that the dynamic random-effects probit model provides the best specification. Our main results indicate that state dependence and unobserved heterogeneity make important contributions to a given health status. We also examine whether the participation in RPA is associated indirectly with a decreased risk for chronic diseases. In section “Conclusions”, we offer conclusions and argue that both participation in RPA and improved health policy are factors that could reduce the costs incurred by Japanese society in the treatment of chronic diseases.

## Participation in physical activity and latent health stock

### Literature review

Several previous studies have used health production models that include participation in physical activity in order to examine the effects of lifestyle choices on health. The results of those studies suggest that individuals with healthier lifestyles tend to have better self-assessed health [9, 10].

Labor force participation should be considered an important factor in health-relevant behavior when we analyze the effects of lifestyle choices on health. First, the kind of work performed has a decisive impact on the health depreciation rate [11, 12]. Blue-collar workers with physically exhausting jobs tend not to exercise after work. Individuals with lower socioeconomic status (SES) are more likely to report engaging in job-related physical activity compared to higher SES individuals, who are more likely to report engaging in leisure-time physical activity [13]. Monthly leisure-time physical activity for males differs significantly among occupations, with clerks having greater physical activity than managers and blue-collar workers [14]. Second, working hours are used to explain the trade-off among work, health investment, and leisure. Long working hours reduce leisure-time and health investment activities. A study of Canadian time-use data collected in 2005 indicated that time poverty may be more important than income poverty as a barrier to RPA. Both income and time deprivation can contribute to low levels of physical activity [15]. Individuals make choices about how to allocate their time and resources to health investments and other activities. On the one hand, time spent on RPA reduces time available for other activities. On the other hand, time spent on RPA increases health stock and in turn reduces time lost to illness. The expenditure of time for health-producing activities such as RPA may improve one’s available hours of productive activity.

Education, employment, and income are among the most powerful components of SES, and lower levels of education can lead to insecure income, hazardous work conditions, and poor housing, which can in turn increase the risk of death due to external causes [16]. Educational attainment has been shown to have a positive association with habitual exercise regardless of age [17].^5^ Several important behavioral risk factors for poor health are more common among people in lower SES groups. Nishi et al. [18] reported that females with lower educational levels were more likely to have a smoking habit.

Adults with lower incomes or less education are more likely to smoke and more likely to be obese than adults with higher incomes and more education. Low-income individuals tend to consume cheaper meals with lower nutritional value. As a consequence, the risk of overweight or even obesity is much higher for those with low incomes.

In contrast, individuals at the highest levels of income, education, and job classification were more likely to engage in RPA during their leisure time than those with lower job status and incomes. Nonsmoking, moderation in drinking, and normal-range body weight may be seen as the consequences of health investments [19].

The Japanese workplace is characterized by several unique features, such as an intense work environment [16]. On the one hand, hours of market work are likely to affect both income and health. For regular workers, working more hours than the prescribed 40 h per week is a major constraint on leisure-time physical activity. Both higher occupational status and longer working hours may reduce the leisure-time physical activity of middle-aged persons. Managers, for example, tend to have stressful jobs with long working hours that allow less leisure time for disease-preventive physical activity. On the other hand, increases in non-market working time make it less costly to undertake health-conducive activities such as exercise or the consumption of a healthy diet [20].

### Data

Table 1 shows a summary of statistics for all health-related variables at an individual level. The data were obtained from nationwide surveys in Japan. The 5 years’ longitudinal data (2005–2009) used in this study were taken from the Longitudinal Survey of Middle and Elderly Persons (LSMEP) by the Japanese Ministry of Health, Labor and Welfare. The respondents of this survey were 50–59 years old in 2005. Data were collected through a combination of interviews and self-administered questionnaires. The LSMEP asked each respondent about his or her illnesses and lifestyle variables. From these surveys, we obtained information about demographic variables, educational background, and occupational status. However, the LSMEP did not ask about the number of family members or the number of housemates, except for spouses.

**Table 1:** Descriptive statistics

Variables	Males		Females		All	
	*N* = 52,743		*N* = 40,858		*N* = 93,601	
	Mean	SD	Mean	SD	Mean	SD
*1. Lifestyle*						
Dummy for light regular physical activity	0.137	0.34	0.179	0.38	0.155	0.36
Dummy for moderate regular physical activity	0.154	0.36	0.149	0.36	0.152	0.36
Dummy for vigorous regular physical activity	0.023	0.15	0.030	0.17	0.026	0.16
Dummy for light irregular physical activity	0.055	0.23	0.078	0.27	0.065	0.25
Dummy for moderate irregular physical activity	0.108	0.31	0.089	0.28	0.100	0.30
Dummy for vigorous irregular physical activity	0.041	0.20	0.046	0.21	0.043	0.20
Dummy for current smoker	0.436	0.50	0.125	0.33	0.300	0.46
Dummy for heavy smoking	0.204	0.40	0.020	0.14	0.123	0.33
Dummy for almost every day or every day drinker	0.177	0.38	0.351	0.48	0.253	0.43
Dummy for overconsumption of alcohol	0.046	0.21	0.070	0.26	0.056	0.23
*2. Demographic variables*						
Age	56.59	3.09	56.37	3.06	56.49	3.08
Married	0.822	0.38	0.803	0.40	0.814	0.39
Divorced or widowed	0.011	0.10	0.029	0.17	0.019	0.14
Other	0.167	0.37	0.168	0.37	0.167	0.37
*3. Objective health status (doctor’s diagnosis)*						
Dummy for diabetes	0.116	0.32	0.055	0.23	0.090	0.29
Dummy for heart disease (angina, myocardial infarction)	0.048	0.21	0.021	0.14	0.036	0.19
Dummy for cerebral stroke	0.016	0.12	0.008	0.09	0.012	0.11
Dummy for hypertension	0.266	0.44	0.208	0.41	0.241	0.43
Dummy for hyperlipemia	0.136	0.34	0.137	0.34	0.136	0.34
Dummy for cancer	0.015	0.12	0.018	0.13	0.016	0.13
*4. Mental health (during the past 30* *days, how often did you feel…; 1* = *all the time)*						
Dummy for nervous	0.017	0.13	0.026	0.16	0.021	0.14
Dummy for hopeless	0.007	0.08	0.008	0.09	0.007	0.09
Dummy for restless or fidgety	0.006	0.08	0.007	0.08	0.006	0.08
Dummy for so depressed nothing could cheer you up	0.009	0.09	0.013	0.11	0.011	0.10
Dummy for that everything was an effort	0.009	0.09	0.011	0.10	0.010	0.10
Dummy for worthless	0.007	0.08	0.009	0.09	0.008	0.09
*5. Use of medical care and difficulty due to a physical health problem*						
Dummy for medication or doctor’s consultation	0.308	0.46	0.241	0.43	0.279	0.45
Dummy for hospitalization during the past year	0.020	0.14	0.010	0.10	0.016	0.13
Dummy for difficulty in daily life activities	0.056	0.23	0.084	0.28	0.068	0.25
*6. Household characteristics (RI: 10 thousands yen per month at the time of the survey)*						
Dummy for ln(1 + real income): low (RI < 12.98)	0.068	0.25	0.542	0.50	0.275	0.45
Dummy for ln(1 + real income): middle (12.98 ≤ RI < 26.38)	0.235	0.42	0.282	0.45	0.256	0.44
Dummy for ln(1 + real income): high (26.38 ≤ RI < 41.94)	0.332	0.47	0.113	0.32	0.236	0.42
Dummy for ln(1 + real income): very high (41.94 ≤ RI)	0.365	0.48	0.064	0.24	0.233	0.42
Dummy for housemates except spouse	0.708	0.45	0.676	0.47	0.694	0.46
Dummy for care for family members	0.078	0.27	0.120	0.33	0.096	0.30
Dummy for child care for family members	0.036	0.19	0.084	0.28	0.057	0.23

*Sources*: *Longitudinal Survey of Middle and Elderly Persons* 2005, 2006, 2007, 2008, and 2009

Real income does not include income from public pensions. Heavy smoking is a dummy variable for smoking more than 20 cigarettes per day. Overconsumption of alcohol is a dummy variable for the quantity of the alcohol more than the beer of a large-sized bottle per day

As lifestyle variables, we converted the survey responses into dichotomous variables (yes = 1) for each of the following: regular physical activity (RPA), consumption of alcoholic beverages almost every day or every day, and current smoking. We used RPA as a dummy variable, which took on the value of one if individuals who engage in RPA were defined as taking part in sports more than twice a week during their leisure time. The intensity of exercise was classified as follows: light (stretch, light gymnastics), moderate (walking, jogging), or vigorous (aerobics, swimming). The proportion of respondents who were considered to have participated in RPA was 0.333 (light: 0.155; moderate or vigorous: 0.178). The LSMEP did not ask the amount of time devoted to physical activity.

Each health outcome was measured as a binary variable that took a value of one if the individual reported having any of the following conditions (the proportion of individuals with each condition is given in parentheses): diabetes (0.090), heart disease (0.036), cerebral stroke (0.012), hypertension (0.241), hyperlipemia (0.136), and cancer (0.016). The proportions of individuals who reported feeling the following self-reported mental health statuses all of the time during the past 30 days were: nervous (0.021), hopeless (0.007), restless or fidgety (0.006), so depressed that nothing could cheer you up (0.011), everything was an effort (0.010), and worthless (0.008). The proportion of individuals who were taking medication or had consulted a doctor was 0.279. The proportion experiencing difficulty in activities of daily life because of a physical health problem was 0.068.

Non-market working time includes the time spent on housework and child care, activities that do not generate income but nonetheless affect lifestyle. In Japan, women often specialize in non-market domestic work such as child care, food preparation, and nursing care. Homemakers, unemployed persons, and retired persons are not a part of the labor force and are not included in the following analysis. It has been reported that males spend more time doing some type of exercise than females in Japan.^6^ The trend was stable from 1991 to 2006 according to the Survey on Time Use and Leisure Activities published by the Statistics Bureau of the Ministry of Internal Affairs and Communications in Japan [9].^7^ For occupational status, the following proportions were reported: regular employees (0.419), management executives (0.064), part-time and casual workers (0.220), self-employed (0.144), contracted employees (0.067), family workers (0.049), and dispatched workers (0.007). We also employed the data on income per month at the time of the survey. We used income per month of the individuals, because the LSMEP had not asked for household income since 2006.^8^ With respect to demographic variables, we considered age, sex, and marital status. The proportion of married workers was 0.814. Almost half the workers self-reported completing high school. The proportion of workers who self-reported completing a university degree was 0.177.

Real income, which did not include income from public pensions, was deflated by the consumer price index (CPI). The CPI in 2005 was 100. We transformed real income into a natural log, considering the nonlinear association between income and health. The extent of missing income data was relatively larger. Missing data are a major concern for surveys: unless the absence of the variables in question is completely random, the analysis is likely to be biased [21]. We therefore constructed a dummy variable that took on the value of one if the observations had missing values for income. The correlation coefficients between this dummy variable and the relevant health variables are reported in Table 2. All the correlation coefficients shown in Table 2 are very small, and no systematic pattern can be found. We therefore concluded that the missing income data were not systematically related to health variables.^9^

**Table 2:** Missing observations and the correlation between missing and health variables

Missing observations	
Own income	36,680
[1] Males with a spouse’s income did not provide their own income	6,792
[2] Females with a spouse’s income did not provide their own income	22,305
[3] Males (females) with their own income did not provide their own income	15,762
[1] and [3]	4,267
[2] and [3]	3,912

Income^a^	Coefficients of correlation^b^	*p* value	*N*
*Correlation between missing observations and health variables*			
Light regular physical activity	0.013	0.000	146,051
Moderate regular physical activity	0.025	0.000	146,051
Vigorous regular physical activity	0.021	0.000	146,052
Self-assessed health	−0.039	0.000	149,312
Diabetes	−0.005	0.059	128,807
Heart disease	−0.012	0.000	128,671
Cerebral stroke	0.013	0.000	128,566
Hypertension	−0.006	0.031	129,017
Hyperlipemia	−0.010	0.000	128,798
Cancer	0.023	0.000	128,432

^a^A dummy variable in which observations with missing values on income are recoded 1 and all other observations are recoded 0

^b^This column reports the correlation coefficients between the dummy and the relevant health variable

The original self-assessed health (SAH) variable is a six-point scale variable ranging from very good to bad. The SAH variable may be vulnerable to reporting bias because of anticipation and measurement heterogeneity [22]. There may be simultaneity between physical activity and health status, since health affects the participation in leisure-time physical activity directly. In order to overcome the problems associated with the measurement error of SAH, we created a latent health stock variable. To correct for possible reporting heterogeneity, we applied a technique previously proposed by Disney et al. [23].

### Methods

People with worse objective health status may tend to overstate their subjective health. In addition, the self-assessed health status may be affected by personal characteristics such as age, education, or the utilization of medical resources. Following the procedure of [23], we estimated a model of SAH as a function of physical and mental health status ($d_{it}$) as well as personal characteristics such as age and education ($w_{it}$). First, we wrote the unobservable health status ($Z_{it}$) as a function of *d*, *x* and unobserved variables ($\mu_{it}$):

1 $$ Z_{it} = \delta^{\prime } w_{it} + \gamma^{\prime } d_{it} + \mu_{it} . $$

Instead of $Z_{it}$, the categorical variable SAH ($h_{it}$) was observed in our data set. This variable may be measured with a reporting error since the assessment of health may depend on age, education, and health problems [19]. The latent health stock ($h_{it}^{*}$) as the counterpart of the observed $h_{it}$ is a function of $Z_{it}$ and the reporting error ($e_{it}$) as follows:

2 $$ h_{it}^{*} = Z_{it} + e_{it} . $$

The latent health variable can be linked to the categorical variable $h_{it}$ using the mechanism below:

3 $$ h_{it} = j,\quad if\,\varphi_{j - 1} < h_{it}^{*} < \varphi_{j - 1} ,\quad j = 1,2, \ldots ,\,6. $$

Equation (3) shows that our observable health variable takes the value *j* if the latent health stock lies between the two thresholds $\varphi_{j - 1}$ and $\varphi_{j}$. Combining this observation mechanism with (1), the model can be estimated using an ordered probit model. Using the predicted values, we can normalize the health stock via a z-transformation. We used health stock as a dummy variable, which took on the value of one if the latent health stock was good. It was classified according to the median of the standardized variable (median = good).

Table 3 refers to the estimation results. Eleven health status effects on SAH were significantly negative at the 1 % level. Six illnesses (diabetes, heart disease, cerebral stroke, hypertension, hyperlipemia, and cancer), five mental health variables (nervous, hopeless, worthless, depressed, and everything was an effort), and two health care variables (medication or doctor’s consultation, hospitalization) had negative effects on SAH. Low educational attainment also had negative effects on SAH. In contrast, greater education had positive impacts on SAH at the 1 % significance level.

**Table 3:** Estimation results: self-assessed health (*N* = 79,167)

Dependent variable: self-assessed health	
Age	0.0211*** (0.00127)
*Educational attainment*	
Junior high school	−0.149*** (0.0113)
Vocational school	0.0251* (0.0145)
Junior college or technical college	0.0769*** (0.0156)
University	0.124*** (0.0104)
Graduate school	0.290*** (0.0357)
Other educational attainment	−0.0933* (0.0515)
*Health care use*	
Medication or doctor’s consultation	−0.262*** (0.0133)
Hospitalization	−0.542*** (0.0313)
*Health status*	
Difficulty in daily life activities	−0.916*** (0.0159)
Nervous	−0.588*** (0.0308)
Hopeless	−0.158*** (0.0595)
Restless or fidgety	0.0529 (0.0620)
Depressed	−0.402*** (0.0505)
Everything was an effort	−0.448*** (0.0479)
Worthless	−0.253*** (0.0529)
Diabetes	−0.530*** (0.0146)
Heart disease	−0.490*** (0.0213)
Cerebral stroke	−0.327*** (0.0356)
Hypertension	−0.226*** (0.0128)
Hyperlipemia	−0.218*** (0.0118)
Cancer	−0.618*** (0.0325)
Pseudo *R* ^2^ = 0.0764	
Log likelihood = −93,872.859	
LR *χ* ^2^ (22) = 15,539.09	
Prob > *χ* ^2^ = 0.00	

Standard errors in parentheses

* *p* < 0.1

** *p* < 0.05

*** *p* < 0.01

## Empirical strategy and results

The theoretical notion is that health deteriorates over time but is capable of enhancement as a result of household production. Models of rational decision-making have been developed for a variety of health behaviors. Nevertheless, little is known about the relationship between exercise and health of rational health-capital formation. Becker [24] revealed that there is greater benefit from becoming addicted to activities such as regular exercise if the probability of surviving to older ages is high.^10^ Caputo and Levy [25] showed the effects of an agent’s marginal value of health. If the marginal value of health is negative and exercise is a substitute for consumption and mood, then consumption and work increase while exercise decreases with an agent’s mood state.^11^

Differences in latent mental health may affect participation in leisure-time physical activity and, in turn, affect health stock of the individuals. Healthy workers are more likely to invest in health. In our empirical framework, we assumed that the instantaneous utility function of the individual depended on a lifestyle vector and latent health stock, which was conditional on exogenous variables, and a vector of unobservable factors that influence personal preferences.^12^ When utility was updated with the optimal levels of lifestyle at each period from the utility maximization problem in the previous period, future utility clearly depended on past consumption decisions. Thus, examining the degree of the state dependence of RPA or LHS is important.^13^

### Dynamic random-effects probit model and state dependence

In modeling the state dependence of RPA or LHS among Japanese workers, the analysis begins with the dynamic specification using simple pooled probit specification. Under this formulation, the response probability of a positive outcome depends on the unobserved effect and past experience. It is important to take unobserved heterogeneity into account because ignoring it overestimates the degree of state dependence. Second, the random-effects probit specification allows for unobserved heterogeneity but treats the initial conditions as exogenous. Estimating a standard uncorrelated random-effects probit model implicitly assumes zero correlation between the unobserved effect and the set of explanatory variables.^14^

However, it is reasonable to expect the unobserved effect to be correlated with at least some of the elements of the set of explanatory variables if the unobserved effect captures an individual’s behavior. Therefore, the unobserved effect must be integrated out before estimation can progress [32]. The need to integrate out the unobserved effect evokes the question how the initial observation is to be treated. The treatment of initial conditions of the dynamic random-effects probit model is crucial, since misspecification will result in an inflated parameter of the lagged dependent variable term. Ignoring the initial conditions problem yields inconsistent estimates [32, 33].

Wooldridge proposed a conditional maximum likelihood estimator that considers the distribution conditional on the initial period observations and exogenous covariates. Parameterizing the distribution of the unobserved effects leads to a likelihood function that is easily maximized using preprogrammed commands with standard software [32].^15^ The latent equation for the dynamic random-effects probit model of RPA participation is specified as:

4 $$ y_{it}^{*} = \rho^{\prime } y_{it - 1} + \beta^{\prime } x_{it} + \alpha_{i} + u_{it} , $$

where $y_{it}^{*}$ is the latent dependent variable, $x_{it}$ is a vector of exogenous explanatory variables, $\alpha_{i}$ are individual-specific random effects, and $u_{it}$ are assumed to be normally distributed. The coefficient $\rho$ is the state dependence parameter. The observed binary outcome variable is defined as $y_{it}$ = 1 if $y_{it}^{*}$ ≥ 0 and $y_{it}$ = 0 otherwise. The subscript *i* indexes individuals and *t* time periods.

Following Wooldridge [32], we assume a certain correlation between $x_{\text{it}}$ and $\alpha_{i}$ and therefore the time-averages of all time-varying explanatory variables ($\mathop {x_{i} }\limits^{\_}$) are included in the specification. We implement the conditional maximum likelihood approach by parameterizing the distribution of the individual effects as:

5 $$ \alpha_{i} = \alpha_{0} + \alpha_{1}^{'} y_{i0} + \alpha_{2}^{'} \mathop {x_{i} }\limits^{\_} + \varepsilon_{i} , $$

where $\varepsilon_{i}$ is assumed to be distributed *N*(0, $\sigma_{\varepsilon }^{2}$) and independent of ($y_{i0}$, $\mathop {x_{i} }\limits^{\_}$). $\mathop {x_{i} }\limits^{\_}$ is the average over the sample period of the observations on the exogenous variables. Substituting Eq. (5) into Eq. (4) gives Eq. (6). The estimates of $\alpha_{1}$ are of interest as they are informative about the relationship between the individual effect and initial health. We would expect there to be a positive gradient in the coefficient estimates.^16^

6 $$ y_{it}^{*} = \rho^{'} y_{i,\,t - 1} + \beta^{'} x_{it} + \alpha_{0} + \alpha_{1}^{'} y_{i0} + \alpha_{2}^{'} \mathop {x_{i} }\limits^{\_} \, + \, \varepsilon_{i} + u_{it} $$

### Empirical results

Following [5], we assess the statistical fit of the different models using the Akaike information criteria and Schwarz Bayesian information criteria (AIC and SBIC, respectively) for model selection:

7 $$ {\text{AIC}} = - 2\ln L + 2q $$

8 $$ {\text{SBIC}} = - 2\ln L + (\ln M)q, $$

where *q* represents the number of parameters in each specification and *M* the number of observations. When the estimation results of the three models (pooled probit, random-effects probit, and dynamic random-effects probit) were compared (see Tables 4, 5), both the AIC and SBIC of the dynamic random-effects probit model were the smallest values among the three. The variance of the unobserved individual effect ($\sigma_{u}^{2}$) of the dynamic random-effects probit model of RPA or LHS was significant at 1 %. Therefore, the dynamic random-effects probit model was the best specification. The corresponding pooled probit model is given in the first column of Tables 4 and 5.

**Table 4:** Determinants of regular physical activity

Estimation	Pooled probit	Random-effects probit	Dynamic random-effects probit
Dependent variable	RPA	RPA	RPA
Regular physical activity (*t* − 1)	1.426*** (0.0129)	1.426*** (0.0129)	0.608*** (0.0266)
Latent health stock (*t* − 1)	0.0646*** (0.0129)	0.0646*** (0.0129)	0.0539*** (0.0179)
ΔMedication or doctor’s consultation	0.0750*** (0.0223)	0.0750*** (0.0223)	0.0829*** (0.0280)
Low income	0.00525 (0.0229)	0.00525 (0.0229)	0.0244 (0.0328)
High income	0.00753 (0.0199)	0.00753 (0.0199)	0.0131 (0.0282)
Very high income	0.0548** (0.0218)	0.0548** (0.0218)	0.0526* (0.0314)
Lehman shock dummy from middle income to low income	0.0579 (0.0659)	0.0579 (0.0659)	0.0587 (0.0895)
Lehman shock dummy from high income to low income	0.0617 (0.138)	0.0617 (0.138)	0.149 (0.181)
Lehman shock dummy from very high income to low income	−0.00239 (0.152)	−0.00239 (0.152)	0.00602 (0.196)
Lehman shock dummy from high income to middle income	0.0927 (0.0623)	0.0927 (0.0623)	0.155* (0.0827)
Lehman shock dummy from very high income to middle income	0.278** (0.112)	0.278** (0.112)	0.342** (0.149)
Lehman shock dummy from very high income to high income	0.0361 (0.0672)	0.0361 (0.0672)	−0.0130 (0.0885)
Current smoker	−0.232*** (0.0152)	−0.232*** (0.0152)	−0.308*** (0.0236)
Almost every day or every day drinker	0.000829 (0.0148)	0.000829 (0.0148)	0.00820 (0.0216)
Sex	0.0348* (0.0191)	0.0348* (0.0191)	0.0613** (0.0296)
Age	0.00689*** (0.00243)	0.00689*** (0.00243)	0.0140*** (0.00383)
Work hours (natural log)	0.347*** (0.124)	0.353*** (0.0885)	0.353*** (0.0885)
Work hours^2^ (natural log)	−0.0909*** (0.0199)	−0.0862*** (0.0142)	−0.0862*** (0.0142)
Commuting time (natural log)	−0.0562** (0.0275)	−0.0299 (0.0182)	−0.0299 (0.0182)
Year	−0.00232 (0.00922)	−0.00232 (0.00922)	
*m* (Δlatent health stock)			−5.522 (12.40)
*m* (current smoker)			−0.0270 (1.291)
*m* [work hours (natural log)]			−2.695 (4.860)
Regular physical activity (Ini)			1.234*** (0.0373)
Constant	−1.241*** (0.205)	−1.241*** (0.205)	7.898 (17.09)
Ln *σ*_*u*_^2^		−10.74*** (3.194)	−0.433*** (0.0592)
*σ*_*u*_		0.0046 (0.0074)	0.8052*** (0.0238)
Intraclass correlation [*σ*_*u*_^2^/(1 + *σ*_*u*_^2^)]		0.00002	0.3933
Likelihood-ratio test of *ρ* = 0 [*χ* ^2^(1)]		0.46 (0.01)	0.00 (676.80)
Log likelihood	−26,097.6	−26,097.6	−22,889.2
AIC	52,299.1	52,301.1	45,890.3
SBIC	52,758.0	52,768.8	46,379.3
*N*	50,253	50,253	45,793

*m*(*X*) means time-average of time-varying explanatory variable (*X*). (Ini) stands for the initial value

Standard errors in parentheses

* *p* < 0.1

** *p* < 0.05

*** *p* < 0.01

**Table 5:** Determinants of latent health stock

Estimation	Pooled probit	Random-effects probit	Dynamic random-effects probit
Dependent variable	LHS	LHS	LHS
Latent health stock (*t* − 1)	1.111*** (0.0122)	1.044*** (0.0160)	0.363*** (0.0239)
Regular physical activity (*t* − 1)	0.105*** (0.0127)	0.110*** (0.0136)	0.103*** (0.0178)
Low income	−0.0484** (0.0221)	−0.0538** (0.0238)	−0.0425 (0.0315)
High income	−0.0206 (0.0190)	−0.0199 (0.0205)	−0.0119 (0.0269)
Very high income	0.0668*** (0.0208)	0.0748*** (0.0226)	0.0825*** (0.0299)
Lehman shock dummy from middle income to low income	−0.0758 (0.0651)	−0.0731 (0.0685)	−0.0977 (0.0891)
Lehman shock dummy from high income to low income	0.137 (0.132)	0.139 (0.139)	0.0595 (0.174)
Lehman shock dummy from very high income to low income	0.246* (0.149)	0.270* (0.156)	0.388** (0.190)
Lehman shock dummy from high income to middle income	−0.0417 (0.0607)	−0.0469 (0.0638)	−0.0845 (0.0795)
Lehman shock dummy from very high income to middle income	0.155 (0.109)	0.156 (0.114)	0.104 (0.145)
Lehman shock dummy from very high income to high income	0.0877 (0.0643)	0.101 (0.0677)	0.0706 (0.0841)
Current smoker	−0.0357** (0.0145)	−0.0413*** (0.0159)	−0.0318 (0.0222)
Almost every day or every day drinker	−0.00983 (0.0143)	−0.0107 (0.0155)	−0.00515 (0.0209)
Sex	−0.0465** (0.0184)	−0.0523** (0.0203)	−0.0338 (0.0286)
Age	0.00589** (0.00233)	0.00650** (0.00258)	0.00831** (0.00368)
Work hours (natural log)	0.222** (0.0892)	0.227** (0.0950)	0.361*** (0.123)
Work hours^2^ (natural log)	−0.0365*** (0.0141)	−0.0375** (0.0151)	−0.0614*** (0.0195)
Commuting time (natural log)	0.00239 (0.0174)	0.00135 (0.0191)	0.00259 (0.0263)
Year	−0.0159* (0.00885)	−0.0197** (0.00923)	
*m* (regular physical activity)			−0.812 (9.760)
*m* (low income)			3.332 (3.270)
*m* (very high income)			4.435 (3.811)
Latent health stock (Ini)			1.039*** (0.0303)
Constant	−1.216*** (0.202)	−1.208*** (0.217)	−3.515 (3.947)
Ln *σ*_*u*_^2^		−2.196*** (0.150)	−0.448*** (0.052)
*σ*_*u*_		0.3335*** (0.0250)	0.7992*** (0.0211)
Intraclass correlation [*σ*_*u*_^2^/(1 + *σ*_*u*_^2^)]		0.1001	0.3898
Likelihood − ratio test of *ρ* = 0 [*χ* ^2^(1)]		0.00 (57.32)	0.00 (676.80)
Log likelihood	−28,763.8	−28,735.2	−25,310.0
AIC	57,629.7	57,574.4	50,730.0
SBIC	58,079.8	58,033.3	51,210.3
*N*	50,253	50,253	45,793

*m*(*X*) means time-average of time-varying explanatory variable (*X*). (Ini) stands for the initial value. Standard errors in parentheses

* *p* < 0.1

** *p* < 0.05

*** *p* < 0.01

Table 4 indicates that the major determinants of participation in RPA were as follows: income, age, educational attainment, work, and lifestyle. Age, high educational attainment (university), special work such as professionals, managerial work, and security had positive effects on RPA participation. In contrast, a smoking habit, low educational attainment, longer work hours, and longer commuting time had negative effects on RPA participation.^17^ We used six Lehman shock dummy variables to capture the effects of a sudden income decrease. The sum total of Lehman shock dummy variables was 0.16 in 2008. One Lehman shock dummy (from very high income to middle income) was statistically significant at the 5 % level, which suggested that some persons with a sudden income decrease changed their lifestyle.

RPA also had positive effects on LHS at the 1 % significance level (see Table 5). We consider that there was a direct causal link between lifestyle choices such as RPA and health stock. Since the estimation results showed that the degree of state dependence of LHS was positive and significant, it would appear that policy interventions that promote RPA have lasting consequences across time.

It is noteworthy that the major determinants of LHS, except RPA, were slightly different from those of RPA (see the third column of Tables 5 and 11). Individuals with higher income had greater LHS. Very high income had positive effects on LHS. Low educational attainment, difficulty in daily life activities, and care for family members had negative effects. The estimation results of the dynamic random-effects probit model, on the contrary, showed that a smoking habit did not have significant effect on LHS. We therefore consider that there were two causal relationships between smoking habit, RPA, and LHS—a flow to RPA from smoking habit and a flow to LHS from RPA.^18^

The intraclass correlation coefficient (ICC) from an error components panel data model is determined as (ICC = $\sigma_{u}^{2} /(1 + \sigma_{u}^{2} )$), where $\sigma_{u}^{2}$ represents the variance of the unobserved individual effect. The ICC measures the proportion of the total unexplained variation that is attributed to the individual effects.

The ICC represents the correlation of health scores across periods of observation. Values of the ICC close to unity indicate high persistence in health outcomes (Jones et al. 2005). We can consider that there existed moderate persistence in RPA because the value of the ICC of RPA was 0.393, which is almost the same as the value of LHS (see Tables 4, 5).

Testing the hypothesis of a non-zero *ρ* is equivalent to testing the presence of true state dependence, having controlled for the unobserved heterogeneity. As the results of the estimation of the dynamic random-effects probit model, the results change substantially and the state dependence estimate was reduced to less than half. As Table 4 shows, the state dependence parameter of RPA, 0.608, was statistically significant at the 1 % level. Past lifestyle is itself a determinant of future lifestyle. The state dependence parameter of LHS, 0.363, was statistically significant at the 1 % level. The size of the estimated coefficient was smaller than that of RPA. The degree of dependence between previous health stock and current health stock exhibited moderate persistence.

The exogeneity of the initial conditions in the dynamic random-effects probit model can be tested by a simple significance test under the null of $\alpha_{1} = 0$ for Eq. (6). As Tables 4 and 5 show, the exogeneity hypothesis was strongly rejected in these models. We therefore concluded that the estimate of the random-effects probit model overstated the extent of state dependence when the unobserved individual-specific effect influenced the initial conditions.

Our main results indicated that state dependence and unobserved heterogeneity were important explanatory factors of a given health status. As a matter of fact, the explanatory power of observed variables vanished when individual-specific effects and lags of the dependent variable were introduced. The variables so affected were marital status (married), work-related variables such as agriculture and forestry fishing, and occupational status (family worker) for the RPA equation, and a smoking habit, low income, high educational attainment (university and graduate school), and occupational status (self-employed, management executive, and domestic side job worker in a home) for the LHS equation. We also found gender differences in the determinants of RPA or LHS (see Table 6).^19^ Very high income had positive effects and longer working hours had negative effects on both RPA participation and LHS in males. For both males and females, it is noted that a smoking habit had negative effects on RPA at the 1 % significance level. Thus, smoking cessation is an important health policy to increase the participation in RPA.

**Table 6:** Gender differences in the determinants of RPA and LHS

Estimation	Dynamic random-effects probit			
	Males	Females	Males	Females
Dependent variable	RPA	RPA	LHS	LHS
Regular physical activity (*t* − 1)	0.630*** (0.0362)	0.578*** (0.0392)	0.137*** (0.0238)	0.0589** (0.0269)
Latent health stock (*t* − 1)	0.0554** (0.0241)	0.0499* (0.0267)	0.361*** (0.0316)	0.363*** (0.0367)
ΔMedication or doctor’s consultation	0.0981*** (0.0365)	0.0615 (0.0438)		
Low income	−0.00509 (0.0649)	0.00885 (0.0393)	−0.0944 (0.0613)	−0.0160 (0.0387)
High income	0.0552 (0.0362)	−0.0513 (0.0483)	−0.0359 (0.0334)	0.0539 (0.0480)
Very high income	0.100** (0.0392)	−0.0638 (0.0612)	0.0873** (0.0364)	−0.00207 (0.0607)
Current smoker	−0.350*** (0.0280)	−0.175*** (0.0466)	−0.0123 (0.0256)	−0.0777* (0.0456)
Almost every day or every day drinker	0.0135 (0.0330)	0.0128 (0.0285)	0.00117 (0.0310)	−0.00997 (0.0284)
Age	0.0178*** (0.00525)	0.00839 (0.00571)	0.00695 (0.00490)	0.0117** (0.00569)
Work hours (natural log)	0.623*** (0.185)	0.0975 (0.178)	0.601*** (0.186)	0.0769 (0.180)
Work hours^2^ (natural log)	−0.128*** (0.0283)	−0.0527* (0.0295)	−0.100*** (0.0280)	−0.0124 (0.0297)
Commuting time (natural log)	−0.0581* (0.0337)	−0.0646 (0.0492)	0.00769 (0.0313)	−0.0183 (0.0492)
*m* (Δlatent health stock)	−8.314 (14.83)	1.639 (23.62)		
*m* (current smoker)	−0.547 (1.636)	0.231 (2.245)		
*m* [work hours (natural log)]	−3.401 (5.783)	−0.301 (9.319)		
Regular physical activity (Ini)	1.285*** (0.0522)	1.165*** (0.0531)		
*m* (regular physical activity)			−10.65 (11.62)	28.83 (19.13)
*m* (low income)			5.482 (3.947)	−4.613 (6.259)
*m* (very high income)			−0.559 (4.580)	17.49** (7.351)
Latent health stock (Ini)			1.073*** (0.0404)	0.993*** (0.0460)
Constant	9.901 (20.33)	−0.0299 (32.78)	0.719 (4.708)	−16.11** (7.726)
Ln *σ*_*u*_^2^	−0.357*** (0.0781)	−0.545*** (0.0916)	−0.440*** (0.0697)	−0.455*** (0.0815)
*σ*_*u*_	0.8365*** (0.0326)	0.7612*** (0.0916)	0.8024*** (0.0279)	0.7962*** (0.0324)
Intraclass correlation [*σ*_*u*_^2^/(1 + *σ*_*u*_^2^)]	0.412	0.367	0.392	0.388
Likelihood-ratio test of *ρ* = 0 [*χ* ^2^(1)]	0.00 (400.06)	0.00 (270.65)	0.00 (495.45)	0.00 (365.81)
Log likelihood	−12,852.5	−9,984.1	−14,659.3	−10,621.8
AIC	25,815.0	20,078.2	29,426.6	21,351.6
SBIC	26,265.4	20,510.7	29,868.8	21,776.2
*N*	26,598	19,195	26,598	19,195

*m*(*X*) means time-average of time-varying explanatory variable (*X*). (Ini) stands for the initial value. Lehman shock dummy variables were included in our model. The other regressors included are the same variables shown in Tables 10 or 11. We could not reject the hypothesis that there was no gender difference in the degree of state dependence at the 5 % significance level. Standard errors in parentheses

* *p* < 0.1

** *p* < 0.05

*** *p* < 0.01

Using a subsample that excluded individuals without RPA, we investigated the effects of the change in the intensity of RPA on LHS.^20^ As Table 7 shows, increasing the intensity of RPA had positive effects on LHS at the 1 % significance level. However, the value of ICC was 0.338, smaller than that of the full-sample estimate 0.389. The coefficient of the previous latent health stock was 0.596, larger than that of 0.363 (see Table 5). The results showed that the full-sample estimate of the coefficient of previous latent health stock was smaller than that of subsample estimate because the former included individuals without RPA. This implies that the individuals with RPA were associated with greater persistence of LHS compared to the individuals without RPA. The impact of individual heterogeneity was reduced (from 0.389 to 0.338) when we excluded individuals without RPA, and unobserved heterogeneity accounted for 34 % of the unexplained variation in LHS.

**Table 7:** Effects of the change in the intensity of RPA on LHS

Estimation	Dynamic random-effects probit		
	All	Males	Females
Dependent variable	LHS	LHS	LHS
Latent health stock (*t* − 1)	0.596*** (0.0335)	0.657*** (0.0440)	0.517*** (0.0519)
Regular physical activity (*t* − 1)	0.145*** (0.0318)	0.122*** (0.0417)	0.175*** (0.0494)
Intensity of regular physical activity (0 = light, 1 = moderate or vigorous)	0.0783*** (0.0276)	0.0605* (0.0363)	0.100** (0.0426)
Low income	−0.0867* (0.0475)	−0.128 (0.0957)	−0.0546 (0.0588)
High income	0.0170 (0.0416)	−0.0173 (0.0528)	0.0712 (0.0718)
Very high income	0.0862* (0.0454)	0.0580 (0.0555)	0.135 (0.0915)
Current smoker	−0.0293 (0.0346)	−0.0149 (0.0389)	−0.0888 (0.0728)
Almost every day or every day drinker	−0.0320 (0.0307)	−0.0435 (0.0456)	−0.0246 (0.0420)
Sex	−0.00410 (0.0412)		
Age	0.000827 (0.00528)	0.00237 (0.00701)	0.00123 (0.00825)
Work hours (natural log)	0.219 (0.181)	0.366 (0.276)	0.0802 (0.259)
Work hours^2^ (natural log)	−0.0372 (0.0293)	−0.0641 (0.0423)	−0.00994 (0.0435)
Commuting time (natural log)	0.00832 (0.0390)	−0.0408 (0.0462)	0.119* (0.0719)
*m* (regular physical activity)	2.580 (15.53)	−17.20 (18.78)	50.70* (30.45)
*m* (low income)	1.137 (5.202)	5.868 (6.360)	−11.93 (9.966)
*m* (very high income)	4.003 (6.063)	−5.824 (7.401)	25.44** (11.67)
Latent health stock (Ini)	0.899*** (0.0427)	0.890*** (0.0560)	0.917*** (0.0665)
Constant	−3.520 (6.276)	5.228 (7.598)	−24.24** (12.29)
Ln *σ*_*u*_^2^	−0.672*** (0.0954)	−0.819*** (0.1374)	−0.513*** (0.1347)
*σ*_*u*_	0.7146*** (0.0341)	0.6637*** (0.0456)	0.7736*** (0.0521)
Intraclass correlation [*σ*_*u*_^2^/(1 + *σ*_*u*_^2^)]	0.338	0.306	0.374
Likelihood-ratio test of *ρ* = 0 [*χ* ^2^(1)]	0.00 (246.00)	0.00 (110.07)	0.00 (135.05)
Log likelihood	−10,313.9	−5,721.1	−4,568.4
AIC	20,739.9	11,550.3	9,244.8
SBIC	21,178.3	11,941.5	9,623.6
*N*	18,572	10,354	8,218

*m*(*X*) means time-average of time-varying explanatory variable (*X*). (Ini) stands for the initial value. Lehman shock dummy variables were included in our model. The other regressors included are the same variables shown in Table 11. We could not reject the hypothesis that there was no gender difference in the degree of state dependence at the 5 % significance level. Standard errors in parentheses

* *p* < 0.1

** *p* < 0.05

*** *p* < 0.01

## Conclusions

No prior investigation has considered the effects of state dependence and unobserved heterogeneity on the relationship between RPA and LHS. Accounting for state dependence corrects the possible overestimation of the impact of socioeconomic factors. We estimated the degree of the state dependence of RPA and LHS among middle-aged Japanese workers. Our dynamic empirical models included RPA as a representative lifestyle choice, on our hypothesis that there is a direct causal link between lifestyle choice and health status. We also included the lagged dependent variables of these two dependent variables in our models and analyzed partial adjustment mechanisms.

The 5 years’ longitudinal data (2005–2009) used in this study were taken from the Longitudinal Survey of Middle and Elderly Persons by the Japanese Ministry of Health, Labor and Welfare. The respondents were subjects who were 50–59 years old in 2005. Because the original self-assessed health variable might be vulnerable to reporting bias, we used health stock as a dummy variable, which took on the value of one if the LHS was good using the procedure of [23].

The dynamic random-effects probit model provided the best specification. The estimate of the random-effects probit model overstated the extent of state dependence when the unobserved individual-specific effect influenced the initial conditions. As the results of the estimation, we found that RPA had positive effects on LHS, taking into consideration the possibility of confounding with other lifestyle variables. These results indicated that there was a direct causal link between RPA and health stock.

There was moderate persistence in RPA. The impact of individual heterogeneity was reduced when we used a subsample that excluded individuals without RPA. In fact, when individuals without RPA were excluded, the unobserved heterogeneity was reduced to 34 % of the unexplained variation in LHS, from 39 % of the unexplained variation in the full-sample estimation result. Increasing the intensity of RPA had positive effects on LHS and caused individuals with RPA to exhibit greater persistence of LHS compared to individuals without RPA. A smoking habit, low educational attainment, longer work hours, and longer commuting time had negative effects on RPA participation. For both males and females, a smoking habit had negative effects on RPA participation at the 1 % significance level. The estimation results showed that the degree of state dependence of LHS was positive and significant and support the implication that policy interventions that promote RPA, such as smoking cessation, have lasting consequences. We therefore concluded that smoking cessation is an important health policy to increase both the participation in RPA and LHS.

Finally, we discuss the main limitation of our empirical analysis. The results of this article would not be applicable to other age groups or for the whole population because there are intergenerational differences in smoking rate and hours worked. For both males and females, their smoking rate in their 30s was higher than in that in their 50s. For males, the proportion of workers who worked long hours was higher in their 30s than in their 50s. For females, the labor force participation rate in their 30s was lower than that in their 50s. The time poverty due to the responsibility for domestic work influences both the participation in the labor market and regular physical activity.

## Appendix

See Tables 8, 9, 10, 11, 12 and 13.

**Table 8:** Descriptive statistics (Table 1 continued)

Variables	Males		Females		All	
	*N* = 52,743		*N* = 40,858		*N* = 93,601	
	Mean	SD	Mean	SD	Mean	SD
*7. Educational attainment*						
Junior high school	0.168	0.37	0.162	0.37	0.165	0.37
High school (reference)	0.466	0.50	0.526	0.50	0.492	0.50
Vocational school	0.054	0.23	0.119	0.32	0.083	0.28
Junior college or technical college	0.027	0.16	0.118	0.32	0.067	0.25
University	0.261	0.44	0.068	0.25	0.177	0.38
Graduate school	0.017	0.13	0.003	0.06	0.011	0.10
Other	0.007	0.09	0.004	0.06	0.006	0.08
*8. Occupational status*						
Self-employed	0.203	0.40	0.067	0.25	0.144	0.35
Family worker	0.006	0.08	0.104	0.31	0.049	0.22
Management executive	0.090	0.29	0.031	0.17	0.064	0.25
Regular employee (reference)	0.551	0.50	0.248	0.43	0.419	0.49
Part-time worker	0.046	0.21	0.445	0.50	0.220	0.41
Dispatched worker	0.007	0.09	0.007	0.08	0.007	0.08
Contracted employee	0.075	0.26	0.056	0.23	0.067	0.25
Domestic side job worker in a home	0.001	0.02	0.017	0.13	0.008	0.09
Other	0.019	0.14	0.022	0.15	0.020	0.14
*9. Work*						
Work hours (natural log)	3.787	0.36	3.415	0.56	3.625	0.49
Commuting time (natural log)	0.634	0.42	0.427	0.31	0.545	0.39
Special work	0.268	0.44	0.163	0.37	0.222	0.42
Managerial work	0.163	0.37	0.027	0.16	0.103	0.30
Office work	0.075	0.26	0.195	0.40	0.127	0.33
Sale	0.082	0.27	0.114	0.32	0.096	0.29
Service (reference)	0.078	0.27	0.204	0.40	0.133	0.34
Security	0.025	0.16	0.001	0.03	0.015	0.12
Agriculture and forestry fishing	0.036	0.19	0.026	0.16	0.032	0.18
Transportation and communication	0.070	0.25	0.006	0.08	0.042	0.20
Production process and labor work	0.141	0.35	0.141	0.35	0.141	0.35
Other	0.001	0.03	0.001	0.04	0.001	0.03
*10. Dwelling*						
Own house (reference)	0.870	0.34	0.864	0.34	0.868	0.34
Rental housing	0.094	0.29	0.108	0.31	0.100	0.30
Company residence	0.019	0.14	0.007	0.08	0.014	0.12
Other residences	0.015	0.12	0.018	0.13	0.016	0.13

*Sources*: *Longitudinal Survey of Middle and Elderly Persons* 2005, 2006, 2007, 2008, and 2009

Most of a domestic side job worker in a home engaged in manual homework

**Table 9:** Dummy variables for Lehman shock *N* = 15,216

	Mean	SD
Dummy for ln(1 + real income): low from middle	0.04	0.20
Dummy for ln(1 + real income): low from high	0.01	0.10
Dummy for ln(1 + real income): low from very high	0.01	0.09
Dummy for ln(1 + real income): middle from high	0.05	0.21
Dummy for ln(1 + real income): middle from very high	0.01	0.11
Dummy for ln(1 + real income): high from very high	0.04	0.19

*Source*: *Longitudinal Survey of Middle and Elderly Persons* 2008

**Table 10:** Determinants of regular physical activity (Table 4 continued)

Estimation	Pooled probit	Random-effects probit	Dynamic random-effects probit
Dependent variable	RPA	RPA	RPA
Married	−0.0688*** (0.0256)	−0.0688*** (0.0256)	−0.0127 (0.123)
Divorced or widowed	−0.839 (0.705)	−0.839 (0.705)	−1.496 (0.912)
Junior high school	−0.0888*** (0.0199)	−0.0888*** (0.0199)	−0.127*** (0.0314)
Vocational school	−0.0133 (0.0244)	−0.0133 (0.0244)	−0.00687 (0.0386)
Junior college or technical college	0.0227 (0.0258)	0.0227 (0.0258)	0.0210 (0.0413)
University	0.0656*** (0.0183)	0.0656*** (0.0183)	0.0924*** (0.0290)
Graduate school	0.0854 (0.0566)	0.0854 (0.0566)	0.0997 (0.0911)
Other educational attainment	0.112 (0.0839)	0.112 (0.0839)	0.177 (0.135)
Difficulty in daily life activities	−0.0276 (0.0261)	−0.0276 (0.0261)	−0.0384 (0.0360)
Housemates except spouse	−0.0715*** (0.0141)	−0.0715*** (0.0141)	−0.0896*** (0.0210)
Care for family members	0.0618** (0.0266)	0.0618** (0.0266)	0.0701* (0.0365)
Child care for family members	−0.0293 (0.0355)	−0.0293 (0.0355)	−0.0457 (0.0487)
Self-employed	−0.0172 (0.0225)	−0.0172 (0.0225)	−0.0341 (0.0341)
Family worker	0.0745** (0.0363)	0.0745** (0.0363)	0.0382 (0.0549)
Management executive	−0.00973 (0.0268)	−0.00973 (0.0268)	−0.0157 (0.0393)
Part-time worker	−0.0438* (0.0250)	−0.0438* (0.0250)	−0.0470 (0.0367)
Dispatched worker	−0.111 (0.0748)	−0.111 (0.0748)	−0.153 (0.110)
Contracted employee	0.0382 (0.0263)	0.0382 (0.0263)	0.0225 (0.0376)
Domestic side job worker in a home	0.191* (0.113)	0.191* (0.113)	0.225 (0.163)
Other occupational status	−0.00360 (0.0512)	−0.00360 (0.0512)	0.0626 (0.0720)
Special work	0.0785*** (0.0208)	0.0785*** (0.0208)	0.105*** (0.0301)
Managerial work	0.0841*** (0.0265)	0.0841*** (0.0265)	0.121*** (0.0377)
Office work	0.0626*** (0.0230)	0.0626*** (0.0230)	0.0816** (0.0341)
Sale	−0.0229 (0.0250)	−0.0229 (0.0250)	−0.0282 (0.0366)
Security	0.235*** (0.0521)	0.235*** (0.0521)	0.313*** (0.0770)
Agriculture and forestry fishing	−0.114** (0.0471)	−0.114** (0.0471)	−0.115 (0.0712)
Transportation and communication	0.00777 (0.0358)	0.00777 (0.0358)	0.0194 (0.0524)
Production process and labor work	−0.0426* (0.0227)	−0.0426* (0.0227)	−0.0386 (0.0332)
Rental housing	−0.0131 (0.0224)	−0.0131 (0.0224)	0.00407 (0.0348)
Company residence	0.0669 (0.0532)	0.0669 (0.0532)	0.0791 (0.0790)
Other residences	−0.0656 (0.0522)	−0.0656 (0.0522)	−0.0803 (0.0751)

Standard errors in parentheses

* *p* < 0.1

** *p* < 0.05

*** *p* < 0.01

**Table 11:** Determinants of latent health stock (Table 5 continued)

Estimation	Pooled probit	Random-effects probit	Dynamic random-effects probit
Dependent variable	LHS	LHS	LHS
Married	−0.0369 (0.0247)	−0.0406 (0.0256)	0.362 (0.231)
Divorced or widowed	0.541 (0.519)	0.577 (0.543)	6.413 (695.3)
Junior high school	−0.0585*** (0.0189)	−0.0654*** (0.0209)	−0.102** (0.0465)
Vocational school	0.0179 (0.0235)	0.0208 (0.0259)	0.0341 (0.0489)
Junior college or technical college	0.0421* (0.0251)	0.0458* (0.0278)	0.0906* (0.0477)
University	0.0675*** (0.0175)	0.0757*** (0.0195)	0.0988 (0.0620)
Graduate school	0.147*** (0.0552)	0.164*** (0.0615)	−0.0688 (0.253)
Other educational attainment	−0.153* (0.0833)	−0.158* (0.0915)	−0.584** (0.266)
Difficulty in daily life activities	−0.714*** (0.0302)	−0.760*** (0.0327)	−0.845*** (0.0582)
Housemates except spouse	−0.0182 (0.0136)	−0.0179 (0.0148)	−0.0214 (0.0309)
Care for family members	−0.141*** (0.0262)	−0.152*** (0.0280)	−0.203*** (0.0502)
Child care for family members	−0.0182 (0.0344)	−0.0215 (0.0368)	−0.0473 (0.0622)
Self-employed	0.0796*** (0.0213)	0.0891*** (0.0233)	0.0872 (0.0685)
Family worker	0.000201 (0.0351)	0.00643 (0.0383)	0.00614 (0.0611)
Management executive	0.0809*** (0.0258)	0.0867*** (0.0281)	0.0602 (0.0841)
Part-time worker	0.0291 (0.0241)	0.0328 (0.0261)	0.0539 (0.0465)
Dispatched worker	0.0895 (0.0715)	0.0936 (0.0771)	−0.00256 (0.161)
Contracted employee	0.0314 (0.0253)	0.0376 (0.0274)	0.128** (0.0616)
Domestic side job worker in a home	0.247** (0.110)	0.244** (0.118)	0.233 (0.167)
Other occupational status	0.118** (0.0484)	0.128** (0.0518)	0.238** (0.105)
Special work	0.0348* (0.0200)	0.0368* (0.0216)	0.0659 (0.0462)
Managerial work	0.0176 (0.0256)	0.0198 (0.0276)	0.128 (0.0841)
Office work	0.00408 (0.0224)	0.00565 (0.0243)	0.0572 (0.0427)
Sale	0.0147 (0.0241)	0.0134 (0.0261)	0.0341 (0.0477)
Security	0.0751 (0.0506)	0.0764 (0.0551)	−0.243 (0.446)
Agriculture and forestry fishing	0.0626 (0.0435)	0.0659 (0.0473)	0.181 (0.111)
Transportation and communication	0.0384 (0.0338)	0.0422 (0.0366)	0.187 (0.175)
Production process and labor work	−0.0176 (0.0217)	−0.0184 (0.0235)	0.0128 (0.0452)
Rental housing	−0.00875 (0.0214)	−0.0116 (0.0235)	−0.00995 (0.0491)
Company residence	−0.132** (0.0528)	−0.135** (0.0574)	−0.181 (0.178)
Other residences	−0.0659 (0.0501)	−0.0761 (0.0541)	−0.0863 (0.102)

Standard errors in parentheses

* *p* < 0.1

** *p* < 0.05

*** *p* < 0.01

**Table 12:** Sensitivity analysis: dynamic random-effects probit models for heavy smoking and overconsumption of alcohol

Dependent variable	RPA	LHS
Regular physical activity (*t* − 1)	0.612*** (0.0266)	0.101*** (0.0178)
Latent health stock (*t* − 1)	0.0523*** (0.0178)	0.363*** (0.0239)
ΔMedication or doctor’s consultation	0.0871*** (0.0280)	
Low income	0.0306 (0.0328)	−0.0419 (0.0315)
High income	0.0218 (0.0282)	−0.0101 (0.0268)
Very high income	0.0601* (0.0314)	0.0837*** (0.0299)
Heavy smoking	−0.371*** (0.0320)	−0.110*** (0.0292)
Overconsumption of alcohol	0.0864** (0.0367)	0.0302 (0.0358)
Sex	0.0281 (0.0289)	−0.0234 (0.0280)
Age	0.0152*** (0.00383)	0.00816** (0.00367)
Work hours (natural log)	0.347*** (0.124)	0.360*** (0.123)
Work hours^2^ (natural log)	−0.0908*** (0.0199)	−0.0611*** (0.0195)
Commuting time (natural log)	−0.0596** (0.0275)	0.00221 (0.0263)
*m* (Δlatent health stock)	−5.149 (12.36)	
*m* (current smoker)	−0.0373 (1.288)	
*m* [work hours (natural log)]	−2.550 (4.843)	
Regular physical activity (Ini)	1.238*** (0.0374)	
*m* (regular physical activity)		−0.997 (9.761)
*m* (low income)		3.385 (3.271)
*m* (very high income)		4.382 (3.812)
Latent health stock (Ini)		1.037*** (0.0303)
Constant	7.279 (17.03)	−3.438 (3.947)

Lehman shock dummy variables were included in our model

*m*(*X*) means time-average of time-varying explanatory variable (*X*). (Ini) stands for the initial value

The other regressors included are the same variables shown in Tables 10 or 11

Standard errors in parentheses

* *p* < 0.1

** *p* < 0.05

*** *p* < 0.01

**Table 13:** Sensitivity analysis: dynamic random-effects probit models for different income class (reference = very high income)

Dependent variable	RPA	LHS
Regular physical activity (*t* − 1)	0.608*** (0.0266)	0.103*** (0.0178)
Latent health stock (*t* − 1)	0.0539*** (0.0179)	0.363*** (0.0239)
ΔMedication or doctor’s consultation	0.0829*** (0.0280)	
Low income	−0.0282 (0.0407)	−0.125*** (0.0391)
Middle income	−0.0526* (0.0314)	−0.0825*** (0.0299)
High income	−0.0396 (0.0274)	−0.0944*** (0.0261)
Current smoker	−0.308*** (0.0236)	−0.0318 (0.0222)
Almost every day or every day drinker	0.00820 (0.0216)	−0.00515 (0.0209)
Sex	0.0613** (0.0296)	−0.0338 (0.0286)
Age	0.0140*** (0.00383)	0.00831** (0.00368)
Work hours (natural log)	0.347*** (0.124)	0.361*** (0.123)
Work hours^2^ (natural log)	−0.0909*** (0.0199)	−0.0614*** (0.0195)
Commuting time (natural log)	−0.0562** (0.0275)	0.00259 (0.0263)
*m* (Δlatent health stock)	−5.522 (12.40)	
*m* (current smoker)	−0.0270 (1.291)	
*m* [work hours (natural log)]	−2.695 (4.860)	
Regular physical activity (Ini)	1.234*** (0.0373)	
*m* (regular physical activity)		−0.812 (9.760)
*m* (low income)		3.332 (3.270)
*m* (very high income)		4.435 (3.811)
Latent health stock (Ini)		1.039*** (0.0303)
Constant	7.950 (17.09)	−3.433 (3.946)

Lehman shock dummy variables were included in our model

*m*(*X*) means time-average of time-varying explanatory variable (*X*). (Ini) stands for the initial value

The other regressors included are the same variables shown in Tables 10 or 11

Standard errors in parentheses

* *p* < 0.1

** *p* < 0.05

*** *p* < 0.01

## Footnotes

1 The numbers of those who have metabolic syndrome and those who are likely to develop metabolic syndrome in the 40–74 population are estimated at about 10.7 million and 9.4 million, respectively.

2 Regular exercisers are defined as those who exercise at least 2 days a week for 30 min or more, for at least 1 year. The proportion of regular exercisers among males was 29.1 %, which was larger than the value of 25.6 % observed in females.

3 Hernández-Quevedo et al. [5] decomposed observed persistence in health outcomes into components attributable to unobserved heterogeneity and state dependence and found that both play important roles in determining health limitations.

4 According to Grossman’s model, we can consider that increases in the depreciation rate over time cause the optimal stock of health to decrease, as the opportunity cost of capital increases.

5 The results revealed that the association between income and habitual exercise was age-dependent: higher income was associated with a higher likelihood of habitual exercise among those aged 25–59 years.

6 The ratio of time spent participating in sports to the sum of market work and housework was two times larger in male workers than female workers in Japan in 2006 [9].

7 Time allocated to sports by males with a job gradually decreased from 1991 to 2006. This tendency was not observed in female workers. Non-working males spent more time participating in sports than male workers, except for men in their forties.

8 Because the LSMEP does not provide any information on the income of other family members, we could not calculate the household equivalent income.

9 It is well known that the sample variance under mean imputation will be seriously underestimated when the response rate is not high.

10 He also suggested that there is causation from better health to better habits since the cost of bad habits is greater for persons in good health.

11 Thayer [26, 27] defined mood as a relatively long-lasting emotional state, which is different from emotion. Mood is less specific, less tense, and less likely to be related to a particular stimulus or event [26].

12 In the model of [25], the influence of the instantaneous utility gap on mood state is integrated into the determination of the time the individual spends to health. The instantaneous utility gap is a difference between the instantaneous utility experienced and the instantaneous utility threshold.

13 By estimating a recursive bivariate probit model without unobserved heterogeneity, we checked the endogenous relationship between RPA and LHS [28]. Such a model is increasingly used when there is an a priori reason to expect a dependent binary variable to be simultaneously determined with a dichotomous regressor [29–31]. There was, however, statistical evidence of a significant negative correlation in the disturbance terms between two equations, which appears to be counterintuitive.

14 When the initial conditions are assumed exogenous, the random effects variance is restricted to zero, implying that there is no unobserved heterogeneity in participation probabilities.

15 We used STATA/SE 12.1. Another solution is the two-step estimation method proposed by Heckman [34], who introduced a set of exogenous instruments. Stewart [35] proposed an application of these estimators in the context of an investigation of the dynamics of the conditional probability of unemployment. He found similar results for both estimators.

16 All time dummies must be dropped from $\mathop {x_{i} }\limits^{\_}$ to avoid perfect collinearity [3]. The contribution of $\alpha_{2}$ will depend on the strength of correlation between the time-invariant regressors and $\alpha_{i}$. As the results of the estimation, most exogenous variables dropped from $\mathop {x_{i} }\limits^{\_}$ because of collinearity.

17 Work types represent the extent to which the work is sedentary. Occupational status does not fully capture the characteristics of sedentary behavior. Sokejima and Kagamimori [36] revealed that there was a *U*-shaped relation between the mean monthly working hours and the risk of acute myocardial infarction. Long working hours including daily commuting time might reduce the time spent for leisure-time physical activity of middle-aged persons.

18 Our two-equation model has a dynamic structure in which one dependent variable (RPA) is used as a lagged independent variable in the other equation for LHS. We also confirmed that there was no estimation bias due to the possible simultaneity of the model, which could give rise to the correlation between the dependent variable of LHS function and the error term of RPA function.

19 We omitted some exogenous variables in Tables 6 and 7 for lack of space. The full version of the tables can be obtained from the authors.

20 During the 22 years of follow-up, Chomistek et al. [37] concluded that vigorous- and moderate-intensity physical activities were associated with lower risk of major chronic disease for 44,551 males aged 40–75 in 1986 and that increasing amounts of vigorous activity remained inversely associated with disease risk.
